# Editorial: Intraoperative Fluorescence Imaging and Diagnosis in Central and Peripheral Nervous System Tumors: Established Applications and Future Perspectives

**DOI:** 10.3389/fonc.2022.845333

**Published:** 2022-02-07

**Authors:** Francesco Acerbi, Morgan Broggi, Constantinos G. Hadjipanayis, Talat Kiris, Karl-Michael Schebesch

**Affiliations:** ^1^ Department of Neurosurgery, Foundation IRCCS Neurological Institute Carlo Besta, Milano, Italy; ^2^ Department of Neurosurgery, Icahn School of Medicine at Mount Sinai, Mount Sinai Health System, New York, NY, United States; ^3^ Department of Neurosurgery, School of Medicine, Koc University, Istanbul, Turkey; ^4^ Department of Neurosurgery, University Medical Center Regensburg, Regensburg, Germany

**Keywords:** CNS tumors, gliomas, PNST, fluorescence, 5-ALA, fluorescein, ICG, confocal endomicroscopy

This Research Topic “*Intraoperative Fluorescence Imaging and Diagnosis in Central and Peripheral Nervous System Tumors: Established Applications and Future Perspectives*” consists of 13 articles contributed by 100 authors in the field of Neurosurgery, Neuropathology, Neuro-oncology, Medical Physics, and Biophotonics. Our aim was to provide a comprehensive and up-to-date understanding of the possible utilization of different intraoperative fluorophores for both diagnostic and therapeutic purposes in the field of neuro-oncological surgery, as well as its combined use with other intraoperative tools. Eight of the 13 published papers were addressing the problem of the utilization of different intraoperative fluorophores for tumor visualization and therapeutic purposes.

Schupper et al. performed a review to describe the most currently used fluorophores for glioma surgery [5-aminolevulenic acid (5-ALA), sodium fluorescein (SF), indocyanine green (ICG)], elucidating the current evidence on the perspective of timing for each. In addition, these authors also mentioned current studies on fluorophores with more directed mechanisms of action, such as tozulesteride (BLZ-100), a conjugate of tumor-specific peptide chlorotoxin paired with a near-infrared fluorophore, Alkylphosphocholine analogs (APCs), small synthetic phospholipid ether molecules targeting specific tumor cells, with different fluorescent spectrum, and Epidermal Growth Factor receptor (EGFR) targeted molecules, conjugated with near-infrared fluorophores. Finally, they also provide a summary of the most common Central Nervous System (CNS) tumors or other pathological conditions that can be operated with fluorescence-guided resection.

Other studies concentrated on the use of 5-ALA in neuro-oncological surgery. Maragkos et al. identified 16 patients with high-grade gliomas (HGG) undergoing fluorescence-guided resection more than 6 hours after 5-ALA administration (in one case 27 hours and 46 minutes after). They showed that all cases had adequate intraoperative fluorescence, without toxicity, suggesting that relaxation on restrictions regarding timing of surgery after 5-ALA administration could have a positive impact on procedure scheduling and workflow, without any additional risks for the patient. Kiesel et al. performed a review on the available literature evaluating the current role, limitations and new approaches of 5-ALA fluorescence during surgery on suspected low-grade gliomas (LGG). They showed that the current 5-ALA technique is limited by the frequent absence of visible fluorescence in pure LGG (i.e. those lesions without any areas of anaplastic transformation), suggesting instead that it could be particularly useful to visualize intratumoral regions with malignant transformation within suspected LGG. Furthermore, they also analyzed possible future approaches to improve intraoperative LGG visualization such as quantitative spectroscopic Protoporphyrin IX (PpIX) measurements, fluorescence lifetime imaging of PpIX, or confocal endomicroscopy.

Four studies focused on the use of SF in CNS tumors and Peripheral Nervous System tumors (PNST). Xu et al. studied the *in vitro* and *ex vivo* optical characteristics of SF to understand its spectroscopic features after biological tissue uptake. They showed that SF exhibits a significant broadening of its emission band together with a batochromic shift after tissue uptake in CNS tumor samples, possibly explained by differences in pH and concentration of the molecule inside the tumoral tissue. Wang et al. discussed the potential role of fluorescein in facilitating targeted supramarginal resection in HGG, by analyzing its strength in tumor identification, also in areas that fail to enhance on T1-weighed MRI, and its limitations. Olguner et al. analyzed the results of fluorescein-guided resection in 49 patients with spinal intradural tumors, 15 being intra- and 34 being extra-medullary. They found a dense and homogenous fluorescence in all extramedullary tumors and in 73.3% of intramedullary tumors, while 13.3% of intramedullary tumors presented lower and more heterogeneous fluorescence. The use of SF was considered helpful to remove the lesion in 95.9% of the cases. Pedro et al. studied the application of low-dosage of SF for fluorescence-guided resection or biopsy of PNST, analyzing a series of 10 patients. The series comprised schwannomas (6 cases), neurofibroma (1 case), malignant PNST (2 cases) and B-cell lymphoma (1 case). In 90% of the cases the surgeon considered the use of SF helpful in performing the surgical procedure. In addition, the authors were able to find specific fluorescence spectra in different histological subtypes and normal nerve fibers by retrospectively analyzing the recorded intraoperative pictures.

Raspagliesi et al. evaluated the feasibility and safety of sonodynamic therapy in a preclinical animal model using both 5-ALA and SF as sonosensitizers. In particular, they showed that it was safe to sonicate the entire brain parenchyma after administration of sonosensitizing agents, demonstrating that no grey or white matter areas were subjected to macro- and microscopic damage.

Five articles focused on the use the confocal laser endomicroscopy (CLE) in neuro-oncological surgery, using SF. Acerbi et al. reported the results of a prospective *ex vivo* study enrolling 15 patients operated on for primary glioblastomas (GBM) with fluorescein-guided technique and using the CONVIVO^®^ system, a new miniature CLE available on the market. Several biopsies both at the tumor central core and at the tumor margins were performed and analyzed by the CONVIVO^®^ system and then submitted for frozen and permanent sections. A blind comparison between confocal images and frozen/permanent section results was then performed. This comparison showed that the CONVIVO^®^ system was revealed to be a quick (mean time for image interpretation was 5.74 minutes) and reliable method to recognize GBM characteristics at the tumor core, raising the possibility to use it as a complementary tool for intraoperative diagnosis. The research group at the Barrow Neurological Institute greatly contributed to this field by presenting three papers. In the paper by Belykh et al., they confirmed the high specificity (90% overall, 94% in gliomas) and positive predictive value of *ex vivo* tissue analysis with CLE in 47 patients (122 biopsies) affected by different brain tumors advocating its early shift to an *in vivo* use. They also introduced the idea of a second SF injection (reported in 7 patients) to improve image quality and consequently CLE diagnostic accuracy. This concept was further investigated by Abramov et al.: they compared the CLE images of six patients in which a second dose of SF was administered during surgery, according to the neurosurgeon’s request, because the SF signal did not sufficiently yield clear, interpretable, actionable CLE images, with those of the first initial dose of the same group of patients and with those of another nine patients in which a single dose of SF was used. The article’s conclusions were that, when judged necessary especially in prolonged procedures, redosing SF improves the CLE image quality and therefore its usefulness without side effects. Finally, following the very same concept, Belyck et al. reported the application of a high dose of SF (40 mg/Kg) and CLE in a case of a suspected LLG, without contrast-enhancing on preoperative Magnetic Resonance (MR). Very clear and nice images were obtained from the CLE system and were suggestive for a grade 2/3 glioma, confirmed by frozen section; final histology revealed a grade 3 anaplastic oligodendroglioma. The authors encouraged further investigation of this application in non-enhancing “borderline” gliomas. Lastly, in their very interesting paper, Ziebart et al. tried to overcome operator-dependent images interpretation errors by training two different machine learning systems to analyze *ex vivo* 13.972 CLE images obtained from 25 patients with different brain tumors: this residual network model was able to provide automated, real-time analysis of tumors specimen based on CLE images dataset. Once again, *in vivo* studies were warranted to further assess the intraoperative advantages of CLE technique.

Intraoperative tumor visualization represents one of the most important problems in neuro-oncological surgery. The use of intraoperative fluorescence, as also shown by the articles published in this Research Topic, represents one of the most interesting and effective ways to improve the macroscopic and microscopic discrimination between tumoral and non-tumoral tissue at the tumor margin, with a possible impact on patients’ prognosis.

## Author Contributions

FA, MB, CH, TK, and KM-S analyzed the papers published in the Research Topic and contributed to the Editorial.

## Conflict of Interest

FA and KM-S received fees from Carl Zeiss Meditec for lectures at International Scientific Meetings. CH is a consultant for NX Development Corporation (NXDC) and Synaptive Medical. NXDC, a privately held company, markets Gleolan (5-ALA, aminolevulinic acid hydrochloride). Gleolan is an optical imaging agent approved for the visualization of malignant tissue during glioma surgery. CH is a consultant for NXDC and receives royalty payments for the sale of Gleolan, has also received speaker fees by Carl Zeiss and Leica.

The remaining authors declare that the research was conducted in the absence of any commercial or financial relationships that could be construed as a potential conflict of interest.

## Publisher’s Note

All claims expressed in this article are solely those of the authors and do not necessarily represent those of their affiliated organizations, or those of the publisher, the editors and the reviewers. Any product that may be evaluated in this article, or claim that may be made by its manufacturer, is not guaranteed or endorsed by the publisher.

